# A stress inducible SUMO conjugating enzyme gene (*SaSce9*) from a grass halophyte *Spartina alterniflora* enhances salinity and drought stress tolerance in *Arabidopsis*

**DOI:** 10.1186/1471-2229-12-187

**Published:** 2012-10-10

**Authors:** Ratna Karan, Prasanta K Subudhi

**Affiliations:** 1 School of Plant, Environmental, and Soil Sciences, Louisiana State University Agricultural Center, 104 Sturgis Hall, Baton Rouge, LA, 70803, USA

**Keywords:** Abiotic stress tolerance, Halophyte, Stress inducible, SUMO conjugating gene, *Spartina alterniflora*

## Abstract

**Background:**

SUMO (Small Ubiquitin related Modifier) conjugation is a post translational regulatory process found in all eukaryotes, mediated by SUMO activating enzyme, SUMO conjugating enzyme, and SUMO ligase for the attachment of SUMO to its target protein. Although the mechanism for regulation of SUMO conjugation pathway genes under abiotic stress has been studied to certain extent, the role of SUMO conjugating enzyme in improving abiotic stress tolerance to plant is largely unexplored. Here, we have characterized a SUMO conjugating enzyme gene ‘*SaSce9’* from a halophytic grass *Spartina alterniflora* and investigated its role in imparting abiotic stress tolerance.

**Results:**

*SaSce9* gene encodes for a polypeptide of 162 amino acids with a molecular weight of ~18 kD and isoelectric point 8.43. Amino acid sequence comparisons of *SaSce9* with its orthologs from other plant species showed high degree (~85-93%) of structural conservation among each other. Complementation analysis using yeast SCE mutant, *Ubc9*, revealed functional conservation of *SaSce9* between yeast and *S. alterniflora. SaSce9* transcript was inducible by salinity, drought, cold, and exogenously supplied ABA both in leaves and roots of *S. alterniflora.* Constitutive overexpression of *SaSce9* in *Arabidopsis* through *Agrobacterium* mediated transformation improved salinity and drought tolerance of *Arabidopsis*. *SaSce9* overexpressing *Arabidopsis* plants retained more chlorophyll and proline both under salinity and drought stress. *SaSce9* transgenic plants accumulated lower levels of reactive oxygen under salinity stress. Expression analysis of stress responsive genes in *SaSce9 Arabidopsis* plants revealed the increased expression of antioxidant genes, *AtSOD* and *AtCAT,* ion antiporter genes, *AtNHX1* and *AtSOS1,* a gene involved in proline biosynthesis, *AtP5CS,* and a gene involved in ABA dependent signaling pathway, *AtRD22.*

**Conclusions:**

These results highlight the prospect of improving abiotic stress tolerance in plants through genetic engineering of the sumoylation pathway. The study provides evidence that the overexpression of *SaSce9* in plant can improve salinity and drought stress tolerance by protecting the plant through scavenging of ROS, accumulation of an osmolyte, proline, and expression of stress responsive genes. In addition, this study demonstrates the potential of the halophyte grass *S. alterniflora* as a reservoir of abiotic stress related genes for crop improvement.

## Background

Plants are constantly challenged by a wide range of environmental stresses such as drought, high salinity, and temperature fluctuations due to their sessile nature. Response to abiotic stresses is very complex, as various stages of plant growth and development can be affected by a particular stress and often several stresses simultaneously [1]. Molecular responses to abiotic stresses include stress perception, signal transduction to cellular components, gene expression, and metabolic changes, which help the plants to adapt to stress environments [2,3].

Post-translational modifications of proteins play an important role in most cellular processes by rapidly altering the functions of preexisting proteins and protein complexes. Sumoylation or SUMO (Small Ubiquitin related Modifier) conjugation is one of the essential post translational regulatory process essentially found in all eukaryotes. It is a three step enzymatic cascade mediated by SUMO activating enzyme (E1 or SAE), SUMO conjugating enzyme (E2 or SCE), and SUMO ligase (E3) for the attachment of SUMO to its target protein [4,5]. SUMO plays an important role in regulation of protein–protein interactions and subcellular locations in yeast and animals [6-8]. Increased level of SUMO conjugates in response to heat shock, oxidative stress, and DNA damaging agents has been reported in mammalian cell cultures [9-12]. Similarly, increased accumulation of SUMO conjugates upon exposure of *Arabidopsis* seedlings to heat shock, H_2_O_2_, ethanol, and amino acid analog canavanine suggests its important role in stress response and protection in plants [13]. Several studies in *Arabidopsis* highlighted the importance of sumoylation in post translational regulation in response to stresses such as salt, cold, drought, heat, copper toxicity, and nutrient deprivation [14-18]. Importance of sumoylation for abiotic stress response is strengthened from the reports on drastic reduction in tolerance to stresses due to mutation of genes involved in SUMO conjugation [14-16,19]. Further, embryo lethality caused by insertional mutation in *AtSAE2* or *AtSCE1* or double mutations in *AtSUMO1* and *AtSUMO2* genes of *Arabidopsis* inferred its role in plant growth and development [17].The reversible conjugation of the SUMO peptide to protein substrates is emerging as a major post-translational regulatory process in plants [16]. *Arabidopsis* SUMO and related enzymes have been implicated in abscisic acid (ABA) responses, flowering time regulation as well as stress responses [20]. In rice (*Oryza sativa),* transcripts for the SCE is regulated by cold, salt, ABA, and heat [21,22]. However, detailed understanding of SCE for abiotic stress tolerance in plant is still in its infancy.

*Spartina alterniflora*, a halophytic grass commonly known as smooth cordgrass, possesses all the known mechanisms of salt tolerance [23]. In this investigation, we have functionally characterized a SCE gene, *SaSce9,* previously obtained from the salinity stressed cDNA library of *Spartina alterniflora*[24]. *SaSce9* is a functional homolog of SUMO conjugating enzyme, *Ubc9* of *Saccharomyces cerevisiae*. Salt, drought, cold, and ABA induced the expression of *SaSce9* in leaves and roots of *Spartina alterniflora*. Furthermore, *SaSce9* overexpression in *Arabidopsis* improved abiotic stress tolerance by regulating the stress responsive genes involved in ion homeostasis, proline accumulation, and detoxification of reactive oxygen radicals. This study demonstrates the potential and superiority of orthologous stress responsive genes from a halophyte grass *Spartina alterniflora* in improving salt and drought stress tolerance in plants.

## Results

### *SaSce9 of spartina alterniflora* is an ortholog of SUMO conjugating enzyme

**Figure 1:**
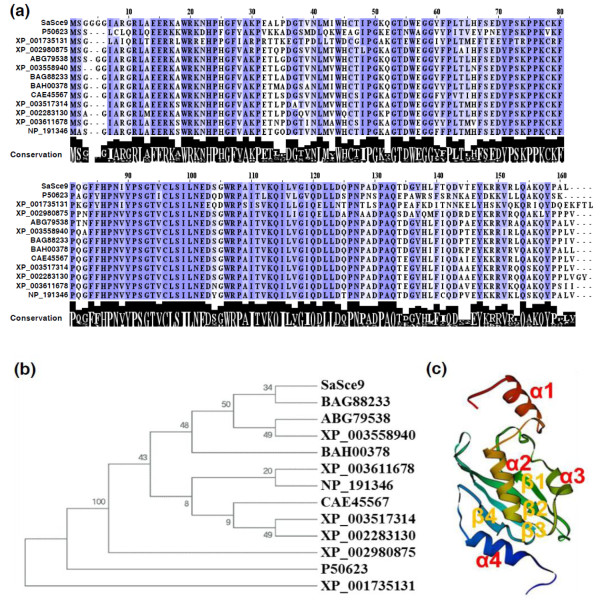
**Multiple sequence alignments, phylogenetic analysis, and predicted tertiary structure of *SaSce9 *protein.** (**a**) Multiple sequence alignment of *SaSce9* protein with SCE proteins from various organisms. Conservation of amino acid residues are shown by black bars below the alignments. Accession numbers of sequences for SCE proteins are: P50623 (*Saccharomyces cerevisiae*), XP_001735131 (*Entamoeba histolytica),* XP_002980875 (*Selaginella moellendorffii*), ABG79538 (*Triticum durum),* XP_003558940 (*Brachypodium distachyon),* BAG88233 {*Oryza sativa* (Os03g0123100)}, BAH00378 {*Oryza sativa* (Os10g0536000)}, CAE45567 (*Nicotiana benthamiana),* XP_003517314 (*Glycine max),* XP_002283130 (*Vitis vinifera),* XP_003611678 (*Medicago truncatula),* NP_191346 (*Arabidopsis thaliana)*; (**b**) Phylogenetic tree of *SaSce9*. The amino acid sequences were subjected to Bootstrap test of phylogeny by the MEGA 4.0 program, using neighbour-joining method with 1000 replicates. Numbers on the Figure are bootstrap values; (**c**) Model of predicted tertiary structure was performed using SWISS-MODEL based on crystallographic data deposited on the Swissprot.

A full length cDNA clone of 489 bp open reading frame, encoding a SUMO conjugating enzyme, named as *SaSce9,* was obtained from the salt stressed cDNA library of *Spartina alterniflora* constructed in our laboratory [24]. *SaSce9* gene codes for a polypeptide of 162 amino acids with a molecular weight of ~18 kD and isoelectric point 8.43. Amino acid sequence comparisons of *SaSce9* with its orthologs from other species showed 90-93% similarity with SCE of monocots such as *Triticum durum*, *Oryza sativa*, *Brachypodium distachyon*, 85-89% to dicots, *Nicotiana benthamiana*, *Glycine max*, *Vitis vinifera*, *Medicago truncatula*, *Arabidopsis thaliana*, 81% to bryophyte *Selaginella moellendorffii*, 53% to *Entamoeba histolytica*, and 57% with *Ubc9* of *Saccharomyces cerevisiae* (Figure. 1a). Clustering of SaSce9 with monocots in phylogenetic analysis and sharing of high degree of homology with counterpart proteins from dicots, yeast, and amoeba, suggested evolutionary conservation of SCE proteins and possible evolution from a common ancestor (Figure. 1b). We also evaluated the conservation of tertiary structure of SaSce9 by modeling of predicted tertiary structure based on crystallographic data deposited on the Swissprot [25]. Comparison of predicted tertiary structure of SaSce9 with that based on crystallographic analysis of the human counterpart HsUbc9 [26], revealed the presence of four alpha helices and four beta sheets in SaSce9 with overall 65% identity with HsUbc9 (Figure. 1c).

### *SaSce9* Functionally complemented *Ubc9* mutant of *saccharomyces cerevisiae*

**Figure 2:**
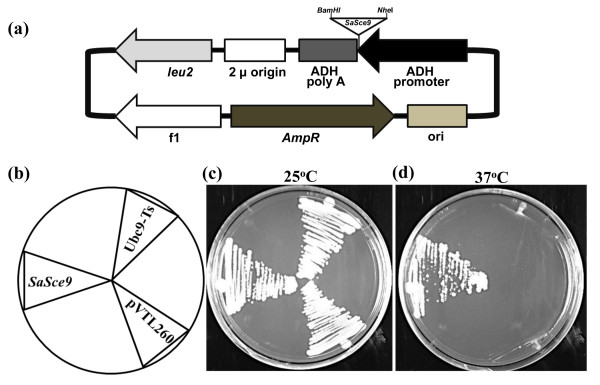
**Complementation of the yeast *Ubc9****-****ts *mutation by expression of the *SaSce9.*** (**a**) Schematic representation of yeast expression vector (pVTL260-*SaSce9*) used for complementation assay, containing an ORF of *SaSce9* cloned at *Nhe*I and *Bam*HI site driven by *ADH* promoter; (**b**) Strains used in this experiment. *Ubc9*-Ts: temperature sensitive mutant for SUMO conjugating enzyme of *Saccharomyces cerevisiae*; pVTL260: *Ubc9*-Ts mutant carrying only vector as control; *SaSce9: Ubc9*-Ts mutant carrying pVTL260-*SaSce9* of *Spartina alterniflora;* (**c**) Growth of yeast strains at 25°C; and (**d**) Growth of yeast strains at 37°C on solid yeast, peptone, and dextrose containing (YPD) media for 3 days.

We analyzed the catalytically conserved role of *SaSce9* using *Ubc9* mutant strain of *S. cerevisiae*. *Ubc9* mutant is temperature sensitive, which normally grows at 25°C but unable to grow at restrictive temperature of 37°C [27]. *SaSce9* ORF was cloned into the yeast expression vector pVTL260 under the regulation of an endogenous alcohol dehydrogenase (ADH) promoter to produce pVTL260-*SaSce9* (Figure. 2a)*,* transformed into yeast *Ubc9* mutant, and grown on synthetic dropout medium lacking leucine, supplemented with 2% glucose at 25°C as well as at 37°C. We found that only pVTL260-*SaSce9* transformed *Ubc9* mutant was able to grow at 37°C, but not the un-transformed or only vector (pVTL260) transformed *Ubc9*. At 25°C, growth of all the strains (Figure. 2b) was normal (Figure. 2c). This experiment indicated the functional complementation of *Ubc9* mutant by *SaSce9* (Figure. 2d).

### Regulation of expression of *SaSce9* by multiple stresses in *spartina alterniflora*

**Figure 3:**
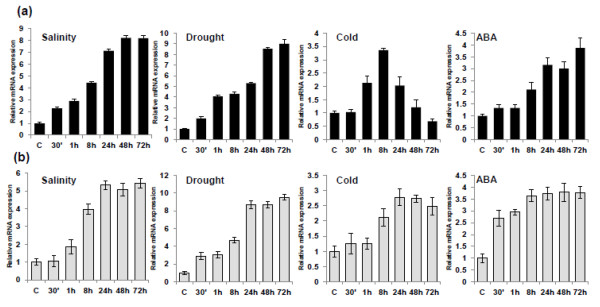
**Expression kinetics of *SaSce9 *in leaves and roots of *Spartina alterniflora *in response to various stresses.** Expression patterns of *SaSce9* at different time intervals in leaves (**a**) and roots (**b**) under salinity stress (5% sea salt), drought (kept on Whatman paper), cold (at 4°C), and ABA (100 μM). Samples were harvested at different time intervals; 30 min, 1 h, 8 h, 24 h, 48 h, and 72 h. Tubulin gene of *Spartina alterniflora* was used as an internal control for normalization of different cDNA samples. Error bars represent standard error of means based on three independent reactions.

Quantitative real-time PCR was used to analyze the expression patterns of *SaSce9* in leaves and roots of *Spartina alterniflora*. As shown in Figure 3, *SaSce9* was constitutively expressed in both leaves and roots of *Spartina alterniflora* but differentially expressed by abiotic stresses such as salt, drought, cold, and ABA. Under salt stress, *SaSce9* expression was gradually upregulated in leaves and roots starting from 30 min up to 72 h of stress. In root, salinity led to highest expression of *SaSce9* within 24 h, whereas in leaves it was observed at 48 h of stress. Under drought stress in root, transcript induction peaked within 24 h, but in leaves it was seen at 48 h of drought stress. Under cold stress, expression in leaves began at 1 h, peaked at 8 h, declines gradually after that, and reached to basal level at 72 h. However, in root, transcript accumulation slightly increased after 30 min, maintained up to 1 h before doubling at 8 h, and reached a maximum peak at 24 h. ABA stress in leaves led to little induction of transcripts up to 1 h, then continued to increase up to 72 h, whereas root showed early and abrupt increase (2.5 times) of transcript accumulation just after 30 min of ABA treatment, then increased up to 8 h and maintained up to 72 h. Increased expression of *SaSce9* transcripts by salinity, drought, cold and ABA revealed the stress responsive nature and possible ABA mediated regulation of *SaSce9* in *Spartina alterniflora.*

### Overexpression of *SaSce9* improved salinity tolerance

**Figure 4:**
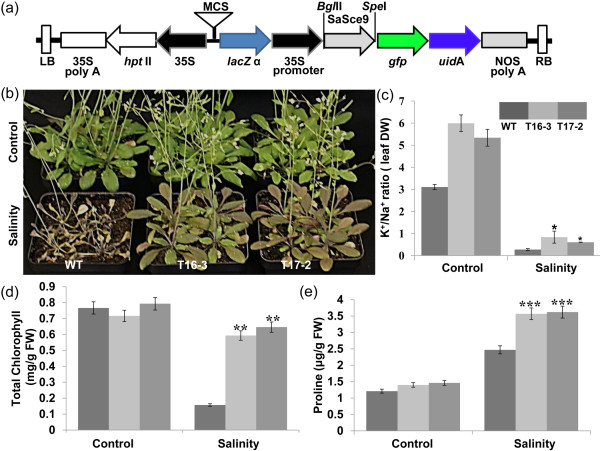
***SaSce9 *overexpressing *Arabidopsis *plants improved salinity tolerance.** (**a**) Schematic representation of pCAMBIA1304 plasmid containing *SaSce9* ORF at *Bgl*II and *Spe*I site driven by cauliflower mosaic virus 35S promoter, used for *Agrobacterium* mediated transformation of *Arabidopsis* using floral dip method; (**b**) Four week-old wild type (WT) and 35S-*SaSce9* transgenic plants grown under normal conditions and salinity stress (150 mM of NaCl) after 20 days of stress. Note that WT plants could not sustain growth under this condition; (**c**) K^+^/Na^+^ ratio based on dry weight (DW) of rosette leaves; (**d**) Chlorophyll content; and (e) Proline content in the rosette leaves of four week-old WT and 35S-*SaSce9* transgenic plants grown under normal conditions after 15 days of 150 mM NaCl stress. Error bars represent standard error of means based on three independent experiments. Comparison was made between WT and individual transgenic lines under control condition or salinity stress by paired t-test. ***, **, and * indicate significant differences in comparison with the control at P < 0.001, P < 0.01 and P < 0.1, respectively. WT represents *Arabidopsis* Columbia ecotype*;* T16-3 and T17-2 represent two independent *SaSce9* transgenic lines. FW: fresh weight of rosette leaves.

To investigate the possible role of *SaSce9* in imparting salinity tolerance in plant, *SaSce9* ORF was cloned into binary vector pCAMBIA1304 under 35S promoter to produce 35S-*SaSce9* (Figure. 4a)*,* and was transformed into *Arabidopsis* ecotype Columbia by floral dip method. T3 homozygous *SaSce9* transgenic plants were analyzed for stress tolerance. Two transgenic lines (T16-3 and T17-2) were chosen for further analysis based on their high level expression of *SaSce9* transcripts (data not shown). Four-week old soil grown wild type (WT) and 35S:*SaSce9* plants were irrigated with 150 mM NaCl until the salt stress induced injury symptoms such as yellowing of rosette leaves and reduced plant heights were visible. As shown in Figure 4b, 35S-*SaSce9 Arabidopsis* plants had less visual salt induced stress injury in comparison to WT plants even after three weeks of stress, while WT plants did not survive. To further evaluate the role of salt stress on ionic balance in 35S-*SaSce9 Arabidopsis*, leaves from WT and transgenic lines were collected after three weeks of salt stress and Na^+^, K^+^ concentration was estimated on dry weight basis. The *SaSce9* transgenic lines, T16-3 and T17-2, showed higher K^+^/Na^+^ ratio under non-stress, and also maintained significantly higher K^+^/Na^+^ ratio than the WT under salinity stress, which indicated the possible role of *SaSce9* in regulating ion homeostasis (Figure. 4c). The *SaSce9* overexpressing transgenic plants accumulated more chlorophyll and proline than the WT under salinity stress (Figure. 4d, e).

### Overexpression of *SaSce9* improved drought tolerance

**Figure 5:**
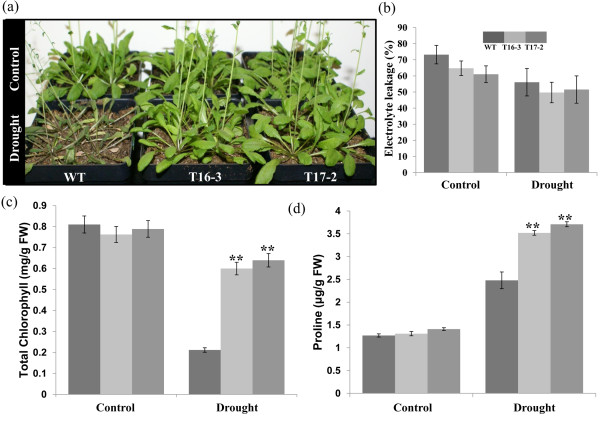
***SaSce9 *overexpressing *Arabidopsis *plants enhanced drought tolerance.** (**a**) Four week-old WT and *SaSce9* transgenic plants after withholding irrigation for 15 days. (**b**) Percent electrolyte leakage; (**c**) Chlorophyll content; and (**d**) Proline content from the rosette leaves of WT and *SaSce9* plants after 7 days of drought. Vertical bar represents mean, and error bar represent standard error of means based on three independent experiments. Comparison was made between wild type and individual transgenic lines under control condition or drought stress by paired t-test. ** indicates significant differences in comparison with the control at P < 0.01. WT represents wild type *Arabidopsis,* T16-3 and T17-2 represent two independent *SaSce9* transgenic lines. FW=Fresh weight of rosette leaves.

To further characterize the function of *SaSce9* for drought tolerance, 4-week-old soil grown WT and *SaSce9* transgenic *Arabidopsis* plants were subjected to water stress for 14 d. At the 14^th^ day of water withholding, most of the WT plants appeared dehydrated and weak, but the *SaSce9* overexpressing plants grew normally and were healthier than the WT (Figure. 5a). To study the membrane stability due to overexpression of *SaSce9*, uniform sized rosette leaves were detached from non-stressed WT and *SaSce9 Arabidopsis* plants and after seven days of continuous drought stress, electrolyte leakage was measured. Electrolyte leakage was relatively less in T16-3, T17-2 plants than WT, under non stress and drought stress (Figure. 5b). Also, chlorophyll levels in *SaSce9* transgenic plants after withholding water for 14 d were significantly higher compared to WT (Figure. 5c). Similarly, the amount of proline after seven days of drought stress was more in T16-3 and T17-2 plants compared to the WT (Figure. 5d).These results showed that overexpression of *SaSce9* improved drought tolerance.

### *SaSce9* Reduced reactive oxygen species (ROS) under drought stress

**Figure 6:**
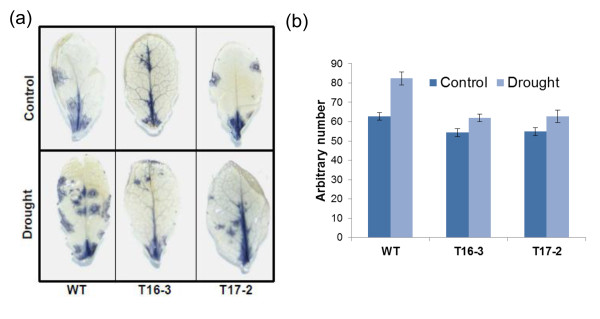
**ROS detection in *SaSce9 *transgenic *Arabidopsis *plants.** The leaves of unstressed and 7 days drought stressed WT and the *SaSce9* transgenic *Arabidopsis* plants were immersed in 1 mg/ml fresh NBT solution, (**a**) Photograph was taken after washing with ethanol, and (**b**) Quantification of stained spots were done using Adobe Photoshop. Experiment was repeated at least three times using rosette leaves from each WT and 35S-*SaSce9* plants and photograph represents one of the three experiments. Vertical bar represents mean and error bar represent standard error of means based on three independent experiments.

We assayed the WT and *SaSce9* transgenic *Arabidopsis* plants for detection of ROS levels using nitroblue tetrazolium (NBT) staining. Leaves from four week old plants after seven days of drought stress and non-stress were incubated into NBT solution. Without drought treatment, leaves of WT and 35S-*SaSce9* transgenic lines, T16-3 and T17-2, showed minimal NBT staining indicating low superoxide levels whereas drought stressed WT plants showed higher level of staining than *SaSce9* overexpressing plants (Figure. 6).

### Expression pattern of stress-responsive genes in *SaSce9* transgenic *Arabidopsis*

**Figure 7:**
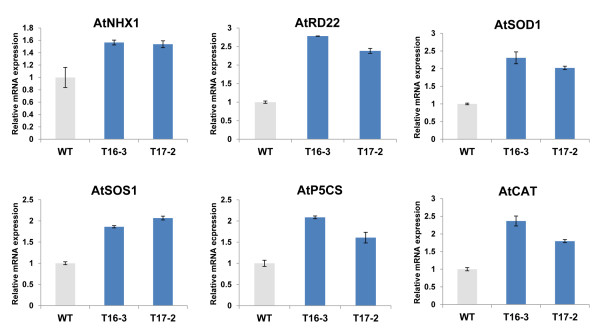
**Expression of stress- responsive genes in *SaSce9 Arabidopsis *plants.** Relative mRNA levels of stress-responsive genes were determined by quantitative RT-PCR using cDNA synthesized from total RNAs isolated from the shoots of 3-week-old *Arabidopsis* plants grown under normal conditions in soil. The *Arabidopsis* tubulin gene was used as an internal control for normalization of different cDNA samples. Error bars represent standard error of means based on three independent reactions.

We further elucidated the possible molecular mechanism of *SaSce9* in stress response by analyzing the expression levels of a set of selected ion transporter genes (*AtNHX1, AtSOS1*), stress responsive genes (*AtP5CS, AtRD22*) and antioxidants genes (*AtSOD1, AtCAT1*) (Table 1) in *SaSce9* transgenic *Arabidopsis* plants. Transcript expression levels for genes belonging to all the three categories were upregulated in *SaSce9* transgenic plants compared to the WT plants (Figure. 7).

**Table 1:** Stress responsive genes and their functions

**Gene**	**Function**	**Reference**
*AtNHX1* (vacuolar Na^+^/H^+^ antiporter)	Compartmentalization of the excessive Na^+^ into vacuole	[28]
*AtSOS1* (plasma membrane Na^+^/H^+^ antiporter)	Na^+^ extrusion from cytosol to surrounding medium	[29]
*AtP5CS* (delta-1-pyrroline-5-carboxylate synthase)	Proline biosynthesis	[30]
*AtRD22* (responsive to dehydration)	Drought stress responsive gene	[31]
*AtSOD1* (superoxide dismutase)	Decomposition of superoxide into hydrogen peroxide	[32]
*AtCAT* (catalase)	Decomposition of hydrogen peroxide into water and oxygen	[33]

## Discussion

Sumoylation pathway is involved in post translational modification of proteins through employment of three key enzymes, SUMO activating enzyme (E1), SUMO conjugating enzyme (SCE or E2), and SUMO ligase (E3) by regulating gene expression, nuclear localization, and signal transduction [13,20]. Sumoylation pathway begins with the activation of the SUMO C-terminus through exposing glycine by a SUMO-specific E1 activating enzyme. Activated SUMO is subsequently transferred to the cysteine residue of a SUMO-conjugating enzyme E2, and then with the help of an E3 ligase, SUMO is finally conjugated to the target lysine residue of a substrate protein [5,34]. In *Arabidopsis,* sumoylation of proteins has been reported in environmental response, flowering time regulation, and phosphorus starvation [13,14,16,19,35-37]. Members of sumoylation pathway such as *AtSCE1* (SUMO conjugating enzyme), *AtSIZ1* (SUMO ligase), AtSUMO1and AtSUMO2 have been identified and found to be involved in ABA signaling and drought response in *Arabidopsis*[14,16,35]. In rice, regulation of SUMO conjugating enzyme genes by temperature stress has also been reported [22]. But whether SUMO conjugating enzyme imparts stress tolerance to plant is still not clear. In this study, we characterized a SUMO conjugating enzyme gene “*SaSce9”* from a halophytic grass, *Spartina alterniflora,* and investigated its role in abiotic stress tolerance mechanisms in plant.

### *SaSce9*, an ortholog of SCE, induced by multiple stresses in halophytic grass *Spartina alterniflora*

We have identified a SUMO conjugating enzyme gene, *SaSce9* from *Spartina alterniflora*, which has same sequence characteristics as the reported yeast SUMO conjugating enzyme, *Ubc9*. The deduced protein sequence analysis of this gene revealed the presence of high structural homology with SCE orthologs from amoeba, yeast, and plants, suggesting evolutionary conserved function of SCE across the eukaryotes. Predicted tertiary structure of *SaSce9* using SWISS-MODEL software suggested the presence of four alpha helices and four beta sheets similar to the known X-ray crystal structure of SCE, HsSce9 of human [26]. To further validate the functionally conserved role of *SaSce9*, a temperature sensitive mutant strain of *Saccharomyces cerevisiae*, *Ubc9*-ts was used [27]. *SaSce9* was able to functionally complement the *Ubc9* mutant of yeast, indicating the potential evolutionary conserved function of *SaSce9* between *Spartina* and yeast. There are several reports for functional complementation of *Ubc9* mutant phenotype by expression of *DmUbc9* from *D. melanogaster*[38], *NbUbc9* from *N. benthamiana*[39], *HsUbc9* from *H. sapiens*[40], and *OsSce1* from *O. sativa*[22].

Sumoylation status of the proteins involved in stress response is reported to be altered as they travel between the nucleus and the cytosol [13,17]. As the *SaSce9* gene was obtained from salinity stressed library of *Spartina alterniflora*, we further wanted to check its organ specific inducibility in leaves and roots of *Spartina alterniflora* under salt, drought, cold, and exogenously supplied ABA stress. Interestingly, the increased expression of *SaSce9* transcript was observed under all the stresses, as shown by qRT-PCR, with highest early induction (30 min) found in leaf by salinity and drought and in root by drought and ABA, suggesting the stress responsive transcript accumulation of *SaSce9* in *Spartina alterniflora*. The inducibility of *SaSce9* gene by ABA indicated that the expression of *SaSce9* may be regulated through ABA responsive pathway in *Spartina alterniflora.* ABA mediates the responses of plant to stress conditions such as salinity, drought, and cold [41,42]. Moreover, salt, cold, and ABA stress induced accumulation of SUMO conjugates and high expression of *AtSCE1a* has been observed in different tissues of *Arabidopsis*[13]. *OsSce1* was found to be induced by temperature stress in the seedlings of rice [22]. Chaikam and Karlson [21] reported developmental and stress responsive regulation of SUMO cascade components with highest level of expression in reproductive tissues suggesting possible role of sumoylation during flowering in rice.

### *SaSce9* enhances salinity and drought tolerance in *Arabidopsis*

Considering the stress inducible response of *SaSce9* in *Spartina alterniflora, SaSce9* gene was further tested for its role *in-planta* towards abiotic stress. Under normal condition, growth and morphology of WT, *SaSce9* transgenic lines were similar. Under 150 mM NaCl stress, both transgenic lines could grow and set seed successfully, while most of the WT plants died. Higher K^+^/Na^+^ ratio in *SaSce9* transgenic lines under non-stress and salinity stress suggested improved basal uptake of K^+^ due to overexpression of *SaSce9.* Several studies have demonstrated the importance of increased K^+^/Na^+^ ratio for tolerance of plants against salinity stress [43-46]. Enhanced expression of two ion transporters, *AtNHX1* and *AtSOS1,* in *SaSce9* transgenic plants compared to WT thus confirmed our hypothesis for the preferential uptake of K^+^ by *SaSce9* transgenic plants*.* Under continuous drought stress, *SaSce9* transgenic plants were healthy, turgid, and green, while WT plants lost its vigor and growth. *SaSce9* expression improved drought tolerance of *SaSce9 Arabidopsis* by maintaining membrane stability as its electrolyte leakage was lower than the WT. Retention of chlorophyll in plants under stress is an important aspect for increased stress tolerance. This study demonstrated that the increased level of chlorophyll under salinity and drought stresses might be an important mechanism for the improvement of stress tolerance due to expression of *SaSce9* in *Arabidopsis.*

### *SaSce9* enhances abiotic stress tolerance through scavenging oxygen radicals and proline production

Proline plays a vital role in maintaining osmotic balance and stabilizing cellular structures in plants [30,47,48]. Increased free proline content in transgenic *Arabidopsis* under salinity and drought stress conditions indicated the role of *SaSce9* in proline accumulation, thereby protecting the plants against the stress. Enhanced expression of *P5CS1* (the key gene involved in proline biosynthesis) in *SaSce9* plants compared to the WT plants further supported our hypothesis for the role of *SaSce9* in osmotic stress tolerance. In an earlier report, transgenic plants overexpressing the *P5CS* gene from *Vigna aconitifolia* accumulated more proline and were more tolerant to osmotic stress [47]. Furthermore, histochemical staining of leaves using NBT showed that even without stress O_2_^-^ could be stained, but the transgenic lines had lower levels of ROS (reactive oxygen species) relative to WT. Drought stress resulted in increased ROS levels in WT but transgenic lines accumulated remarkably less O_2_^-^ as evidenced by the accumulation of less blue products. ROS were produced in both non-stress and stress conditions but the balance between the production and removal of ROS determines its effect on cellular system [30,32,33]. Plants evolve a complex antioxidant system in order to detoxify stress-induced ROS in which ROS scavenging enzymes such as superoxide dismutase and peroxidase play essential role [49]. Increased expression of *AtSOD1* and *AtCAT* in *SaSce9* transgenic plants might be responsible for scavenging ROS produced under drought stress in this study. These analyses further validated the role of *SaSce9* for improving stress tolerance in plant by affecting different stress related pathways. Our observation clearly showed the indirect role of *SaSce9* in scavenging of oxygen radicals resulting in protection of plant against osmotic stress.

## Conclusions

This study demonstrated for the first time that manipulation of a *SaSce9* gene, a member of the sumoylation pathway, through transgenic approach, can lead to improved abiotic stress tolerance in plants. Although, Lois *et al.,*[35] developed *AtSce1a* expressing transgenic *Arabidopsis* plants, the focus of their study was to correlate the level of *AtSce1a* expression with the level of sumoylation which in turn attenuates ABA-mediated growth inhibition and induces ABA- and stress-responsive genes. Thus, we hypothesize that overexpression of *SaSce9* might have increased the sumoylation status in *SaSce9* transgenic plants and improved the stress tolerance by regulating stress responsive genes. Further investigations on identifying interacting partner(s) of sumoylation pathway members and its target protein by comparing SCE mutant would reveal the exact molecular mechanisms for possible role in imparting stress tolerance through manipulation of post translational modifications in plant.

## Methods

### *Spartina alterniflora* plants and stress treatment

Young (three to four-leaf stage) uniform, clonally propagated plants of *Spartina alterniflora* cv. ‘Vermilion’ grown in sand-filled plastic pots under normal growth conditions inside a greenhouse with 14 h light and 10 h dark at 26/18°C day/night temperature were used for stress related experiments [24]. Pots were supplied with Hoagland’s nutrient solution [50]. Salinity stress was imposed by using a 5% (*w*/*v*) solution of commercial synthetic sea salts (Instant Ocean, Aquarium Systems, Mentor, OH, USA) dissolved in Hoagland’s solution, which is equivalent to the salt concentration of sea water (~35 parts per thousand). Drought stress was imposed by keeping uprooted uniform *Spartina* plants on Whatman paper under normal growth condition of greenhouse. For cold stress, pots containing plants were kept at 4°C under dim light. For ABA treatment, plants were supplied with 100 μM ABA (Sigma, USA). Leaf and root tissues were collected at different time intervals of stress i.e. 30 min, 1 h, 8 h, 24 h, 48 h, and 72 h from three representative plants. Both root and leaf tissues were thoroughly washed, wiped with tissue paper, immediately frozen in liquid nitrogen, and stored at −80°C till further use. Unstressed plants were harvested as control.

### Sequence analysis

An expressed sequence tag #968 (EST) of *Spartina alterniflora* from a salinity stressed EST library constructed previously in our laboratory [24] was found to be similar with SUMO conjugate enzyme gene and was referred as ‘*SaSce9’* in this report. It was used for multiple sequence alignment with orthologs from different organisms. Multiple alignments of *SaSce9* were performed using ClustalW program [51] using amino acids, and phylogenetic analyses were conducted in MEGA 4 [52]. Phylogenetic tree of these sequences were inferred using the Neighbor-Joining method [53]. The bootstrap consensus tree inferred from 1,000 replicates was used to represent the evolutionary history of the selected eukaryotic species. The tertiary structure of SaSce9 was predicted by homology modeling based on crystallographic data deposited on the Swissprot using SWISS-MODEL [25].

### Yeast complementation

Complete open reading frame (ORF) of *SaSce9* was PCR amplified using forward primer, *SaSce9Nhe*IF and reverse primer, *SaSce9Bam*HIR containing *Nhe*I and *Bam*HI restriction endonuclease (RE) sites, respectively (Table 1) and ligated into the *NheI* and *Bam*HI cloning site of yeast expression vector pVTL260 to generate pVTL260-*SaSce9* plasmid. The recombinant clone was confirmed by restriction analysis and DNA sequencing. Plasmid pVTL260 obtained from EUROSCARF (Frankfurt, Germany) has *leu2d* as the selectable marker and it uses the yeast *ADH1* promoter and terminator for expression in yeast. For complementation, *Ubc9-ts* temperature sensitive yeast mutant, YWO103 [MAT α ubc9-TRP1, his3-200 leu2-3, 2–112 lys2-801 trp1-1(am) ura3-52 bar1::HIS3] was used [27]. YWO103 is a temperature sensitive mutant of *S. cerevisiae* containing mutant SUMO conjugating enzyme gene, *Ubc9*. YWO103 normally grows at 25°C but is unable to grow at restrictive temperature of 37°C. Transformation of strain YWO103 was carried out as described by Ito *et al.,*[54]. Briefly, yeast cells were grown overnight in YPD medium (BD bioscience, USA) until reaching the mid-log phase (A_600_=1.0), and were then transformed with pVTL260-*SaSce9* or empty pVTL260 (as control) plasmid by PEG/LiCl method. Transformants were selected in a minimal synthetic drop out medium lacking leucine (amino acid used as auxotrophic marker). Transformed yeast cells were checked by PCR for the presence of *SaSce9* gene and colonies were then restreaked onto solid yeast peptone and dextrose (YPD) medium and incubated at permissive (25°C) and restrictive (37°C) temperatures. Cloning of genes was carried out using the protocols in laboratory manual [55]. All the primers used in this study were designed using primer3 input version 4.0 [56].

### RNA isolation and cDNA synthesis

Total RNA was isolated using the RNeasy plant midi kit (Qiagen, USA), and on-column *DNAse*I digestion was carried out to avoid the possible contamination of genomic DNA as per the manufacturer’s instruction (New England Biolab, USA). Quality of total RNA was checked in 1.2% formamide-denaturing agarose gel and quantification was carried out using ND-1000 spectrophotometer (Nanodrop Technologies, USA). First strand cDNA was synthesized using iScript™ first strand cDNA synthesis kit (Bio-Rad, USA) as per the instructions given in the manual.

### Quantitative real time reverse transcription polymerase chain reaction (qRT-PCR)

Quantitative PCR (qRT-PCR) was used in order to evaluate the expression levels of *SaSce9* gene under different stress treatments in the roots and leaves of *Spartina alterniflora.* qRT-PCR reaction was performed following the protocol described by Karan and Subudhi [57]. RNA isolation and cDNA synthesis of the collected samples were performed as mentioned above. Each 10 μl of PCR reaction contained 5 μl 2×SYBR Green mix (Quanta Bioscience, USA), diluted cDNA, and 0.4 μM of each primer, SaSce9RTF and SaSce9RTR, specific for *SaSce9* gene (Table 2), while tubulin gene of *Spartina alterniflora* (Table 2) was used as an internal control for expression normalization in different cDNA samples. Melt curve analysis was performed to check the specificity of amplified product and relative gene expression levels were determined using the 2^-ΔΔCT^ method [58]. The CT (cycle threshold) values for both the target and internal control genes were means of three technical replicates.

**Table 2:** **Primers used for cloning and qRT-PCR of the *SaSce9 *gene**

**Primer name**	**Sequence** (**5**’-**3**’)
SaSce9RTF	TCAGACTGCAGGCTAAGCAG
SaSce9RTR	TGACCCAACGATTTTGTGAA
SaSceBglIIF	GGAAGATCTATGTCTGGGGGTGGGGGAATC
SaSceSpeIR	GGACTAGTTCAGACCAGTGCAGGATACTGCTTAGC
SaSceNheIF	CTAGCTAGCATGTCTGGGGGTGGGGGAATC
SaSceBamHIR	CGGGATCCTCAGACCAGTGCAGGATACTGCTTAGC
pCAMF	GGAGAGAACACGGGGGACTCTTG
SaTUBRTF	GAAGGTGATGAGGGTGATGAGT
SaTUBRTR	TTCAAGCAAACAAGCCTTCATA

The same procedure was followed to analyze the expression patterns of six abiotic stress-related genes (Primers listed in Table 3) in three week old T3 homozygous transgenic *Arabidopsis* and wild type Columbia ecotype plants grown in vermiculite under normal growth conditions. The AtTUBRTF and AtTUBRTR (primers for *Arabidopsis* tubulin gene in Table 3) were used to verify equal concentration of templates for the expression analysis. The qRT-PCR was repeated at least three times, with different number of PCR cycles to confirm the differential expression of stress-related genes in *SaSce9* transgenics.

**Table 3:** **Stress related gene primers used for qRT**-**PCR in *SaSce9 *transgenic *Arabidopsis *plants**

**Primer name**	**Sequence** (**5**’-**3**’)
AtTUBRTF	ATAACCGTTTCAAATTCTCTCTCTC
AtTUBRTR	TGCAAATCGTTCTCTCCTTG
AtRD22F	GATTCGTCTTCCTCTGATCTG
AtRD22R	TGGGTGTTAACGTTTACTCCG
AtP5CSF	GAGGGGGTATGACTGCAAAA
AtP5CSR	AACAGGAACGCCACCATAAG
AtNHX1F	CCGTGCATTACTACTGGAGACAAT
AtNHX1R	GTACAAAGCCACGACCTCCAA
AtSOS1F	TCGTTTCAGCCAAATCAGAAAGT
AtSOS1R	TTTGCCTTGTGCTGCTTTCC
AtSOD1F	TCAACTGGAAATATGCAAGCGAGGT
AtSOD1R	ACCACACAGCTGAGTTGAGCAAA
AtCATF	AGCGCTTTCGGAGCCTCGTG
AtCATR	GGCCTCACGTTAAGACGAGTTGC

### Generation of transgenic plants

The complete ORF of *SaSce9* was amplified by PCR using forward primer SaSce9BglIIF and reverse primer SaSce9SpeIR (Table 1) containing the *Bgl*II and *SpeI* RE sites respectively, with *Pfu* DNA polymerase (NEB, USA). The identity and orientation of p35S-SaSce9 was further confirmed by DNA sequencing. The PCR product was digested with *Bgl*II and *SpeI* and cloned into pCAMBIA1304 vector (CAMBIA, Australia), as *Bgl*II-*Spe*I fragment of *SaSce9* to generate the binary vector 35S-*SaSce9*. The construct harboring 35S-*SaSce9,* was introduced into *Agrobacterium* by freeze thaw method and transferred into wild type Columbia ecotype of *Arabidopsis* by floral dip method [59]. Positive transgenic lines were screened on 40 mg/L hygromycin containing MS medium [60], and integration of transgene was confirmed by PCR using vector specific forward primer, pCAMF and *SaSce9* specific reverse primer, SaSce9SpeIR (Table 2). Expression of *SaSce9* transgene was verified by RT-PCR using cDNA made from total RNA isolated from positive *SaSce9* transgenic plants. The *SaSce9* transgenic plants of T3 generation were further used for salinity and drought stress experiments.

### Salinity and drought tolerance assay

Seeds of WT and *SaSce9* transgenic *Arabidopsis* were sterilized and directly sown on the vermiculite containing potting medium PM-15-13 (Lehle seeds, USA) and kept at 4°C for 4 days then transferred to growth chamber containing white fluorescent light of 100 μmol m^-2^ s^-1^ under 16 h light/8 h dark photoperiod at 23±1°C. Four week-old WT and transgenic plants were further supplied with 150 mM NaCl for 20 days for salinity stress, and water withheld for 14 days were used for drought test. Rosette leaves harvested at different time points were used for various physiological and biochemical assays. At least, three independent experiments with three replicates for each WT and, *SaSce9* transgenic lines were studied.

### Measurement of electrolyte leakage (EL), total chlorophyll content and proline content

Rosette leaves of four week old WT and *SaSce9* transgenic *Arabidopsis*, grown under non-stress and stress conditions for one week, were harvested and used for physiological and biochemical measurements. For EL measurement, protocol of Bajji *et al.,*[61] was used. Briefly, 100 mg leaves were placed in 25 ml distilled water, shaken on a gyratory shaker (200 rpm) at room temperature for 2 h, and the initial conductivity (C1) was measured with a VWR® Traceable® Expanded Range Conductivity Meter (VWR, USA). The samples were then boiled for 10 min to induce maximum leakage. After cooling down at room temperature, electrolyte conductivity (C2) was measured and the relative electrical conductivity (C %) was calculated based on (C1/C2) × 100.

For estimation of total chlorophyll in WT and *SaSce9* lines, protocol suggested by Arnon [62] was followed. About 100 mg of fine powder of leaf tissue was homogenized in 1 ml of 80% acetone and kept for 15 min at room temperature in dark. The crude extract was centrifuged for 20 min at 10000 rpm (rotation per minute) at room temperature, and the resultant supernatant was used for assessing absorbance at 663 and 645 nm with a spectrophotometer (Shimadzu UV-1600, Japan). Total chlorophyll content was computed in terms of fresh weight (FW).

For free proline estimation of WT and *SaSce9* transgenic plants, standard protocol of Bates *et al.,*[63] was followed using fresh leaf tissues. Around 100 mg of tissues were used and extracted in 5 mL of 3% sulphosalicylic acid at 95°C for 15 min. After filtration, 2 mL of supernatant was transferred to a new tube containing 2 mL of acetic acid and 2 mL of acidified ninhydrin reagent. After 30 min of incubation at 95°C, samples were kept at room temperature for 30 min and 5 mL of toluene was added to the tube with shaking at 150 RPM, to extract red products. The absorbance of the toluene layer was determined at 532 nm using spectrophotometer (Shimadzu UV-1600, Japan). Standard curve prepared using different concentration of proline by same method was used for measuring free proline content in experimental samples. The experiment was repeated at least three times.

### Na^+^ and K^+^ estimation

Leaf tissues were harvested from unstressed, salt-stressed plants (three weeks old plants treated with 150 mM NaCl for 20 days) of WT and *SaSce9 Arabidopsis*, and oven-dried at 65°C for 48 h. Fifty milligrams of oven dried tissues were digested with 0.1% HNO_3_ at 100°C for 45 min and then Na^+^ and K^+^ concentrations were measured using inductively coupled plasma-mass spectrometry (ICP-MS, Perkin-Elmer Plasma 400 emission spectrometer).

### In situ histochemical localization of O_2_^-^

For detection of superoxide radicals, histochemical staining with nitro blue tetrazolium (NBT) was followed according to Dong *et al.,*[64] with minor modification. Leaves detached from four week old WT and the *SaSce9 Arabidopsis* plants grown under non-stress or drought stress for next 7 days were vacuum-infiltrated in 1 mg/ml fresh NBT solution (prepared in 10 mM phosphate buffer, pH 7.8) and incubated at ambient temperature until appearance of dark spots. The stained leaves were then bleached in concentrated ethanol, kept in 70% ethanol, and photographed. Images were opened in Adobe Photoshop version 7 (Adobe Systems Incorporated, San Jose, CA) and stained areas of leaves were quantified as described by Lehr *et al*., [65].

### Statistical analysis

Mean values, standard error, and *t*-test were performed with the help of pre-loaded software in Excel, available for statistical calculations ( http://www.Physics.csbsju.edu/stats/t-test.html).

## Abbreviations

ABA: Abscisic acid; ADH: Alcohol dehydrogenase; EL: Electrolyte leakage; ROS: Reactive oxygen species; SCE: SUMO conjugating enzyme; SUMO: Small Ubiquitin related Modifier; WT: Wild type.

## Authors’ contributions

RK and PKS designed the study and wrote the manuscript. RK performed the experiments and analyzed the data. All authors read and approved the final manuscript.
